# Lactic Acidosis: A Novel Presentation of Intravascular Lymphoma

**DOI:** 10.7759/cureus.39201

**Published:** 2023-05-18

**Authors:** Vaishali Deenadayalan, Veena Ganesan, Mihir Shah, Shiraz Fidai, Noah Birch

**Affiliations:** 1 Internal Medicine, John H. Stroger, Jr. Hospital of Cook County, Chicago, USA; 2 Hematology, Rush University Medical Center, Chicago, USA; 3 Pathology, John H. Stroger, Jr. Hospital of Cook County, Chicago, USA; 4 Hematology, John H. Stroger, Jr. Hospital of Cook County, Chicago, USA

**Keywords:** morbidity and mortality, hemophagocytic lymphohistiocytosis (hlh), asian variant, lactic acidosis, intravascular large b-cell lymphoma

## Abstract

Intravascular large B-cell lymphoma (IVLBCL) is a rare form of diffuse large B-cell lymphoma that preferentially grows intravascularly within the capillaries and often has a fatal course. Most of the patients have advanced and disseminated disease at the time of presentation. It is often arduous to make the diagnosis during the antemortem period due to the multitude of presenting symptoms. We report a case of aggressive IVLBCL which presented with a myriad of complaints and acidosis and had a rapid clinical decline.

## Introduction

Intravascular large B-cell lymphoma (IVLBCL) is a rare disorder known to be clinically aggressive and distinguished by the growth of large cells solely within blood vessels of varying calibers. Its presentation is not well characterized, and symptoms are often non-specific, such as fever or altered mental status due to nervous system involvement [1]. In this report, we discuss a patient who presented with striking lactic acidosis and upon further investigation was found to have the Asian variant of IVLBCL and had a rapid clinical decline. Failure to identify its presenting features can lead to significant morbidity and mortality. 

## Case presentation

A male in his early 60s, with a medical history significant for hypertension, insulin-dependent diabetes, and alcohol use, presented with a one-week history of progressive dyspnea and abdominal distension with early satiety associated with generalized weakness, cough, and nausea. He had recently arrived from Southeast Asia.

**Figure 1:**
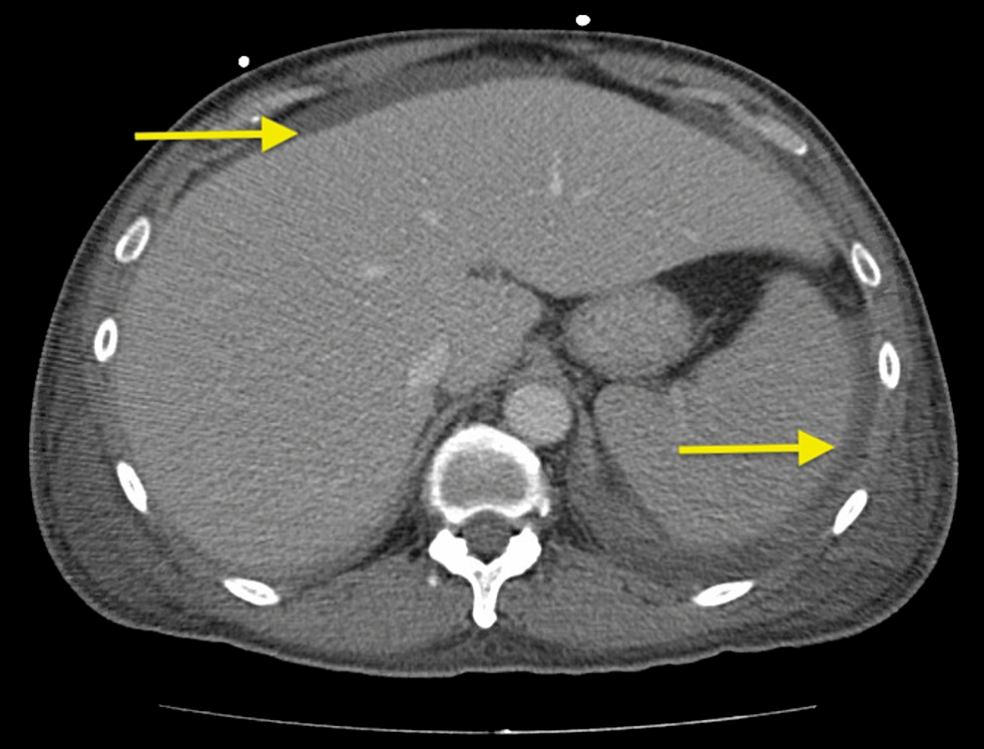
CT abdomen showing hepatosplenomegaly with a small amount of perihepatic and perisplenic free fluid (arrows)

On presentation to the emergency room, he was in moderate distress with tachypnea and was hemodynamically stable. His labs were significant for hyperkalemia, elevated creatinine, cholestatic liver function test elevation with a disproportionately elevated lactate dehydrogenase (LDH) of 1,160, and a notable anion gap metabolic acidosis (anion gap of 26, bicarbonate of 11, and lactate of 12.8). He was also noted to have hepatosplenomegaly as depicted in Figure 1. He was subsequently admitted to the medical intensive care unit (MICU) for hemodialysis as his lactate did not improve with hydration. His symptoms were initially attributed to alcohol use disorder with hypovolemia and thiamine deficiency versus possible metformin-induced lactic acidosis as the leading differentials.

His lactate continued to be persistently elevated despite the initiation of intermittent hemodialysis and was switched to continuous venovenous hemofiltration (CVVH). Over the course of his admission, his lactate did not improve after CVVH, which decreased the suspicion of metformin-induced lactic acidosis. Other common causes of lactic acidosis such as sepsis, hypoperfusion, and mesenteric ischemia were eventually ruled out. The patient was further found to have normocytic anemia with elevations in his reticulocyte index and LDH with hyperferritinemia along with significant thrombocytopenia. These lab findings and imaging indicating hepatosplenomegaly led to a hematology consult for suspicion of a lymphoproliferative process causing a type B lactic acidosis. 

A peripheral smear was reviewed by the hematopathology team, which showed occasional large, atypical lymphoid cells in circulation comprising cells with a high nucleus-to-cytoplasmic ratio, partial clumped chromatin, prominent nucleoli, and agranular deeply basophilic cytoplasm. This raised concern for peripheral blood involvement by mature lymphoma cells. Flow cytometry performed on the peripheral blood showed leucocytosis with mature large B cells positive for CD19, CD20, and CD5, anemia, and severe thrombocytopenia with nucleated RBCs (13/100 cells). He then underwent an emergent bone marrow biopsy and liver biopsy as there was a strong suspicion of type B lactic acidosis due to lymphoproliferative disorder. 

**Figure 2:**
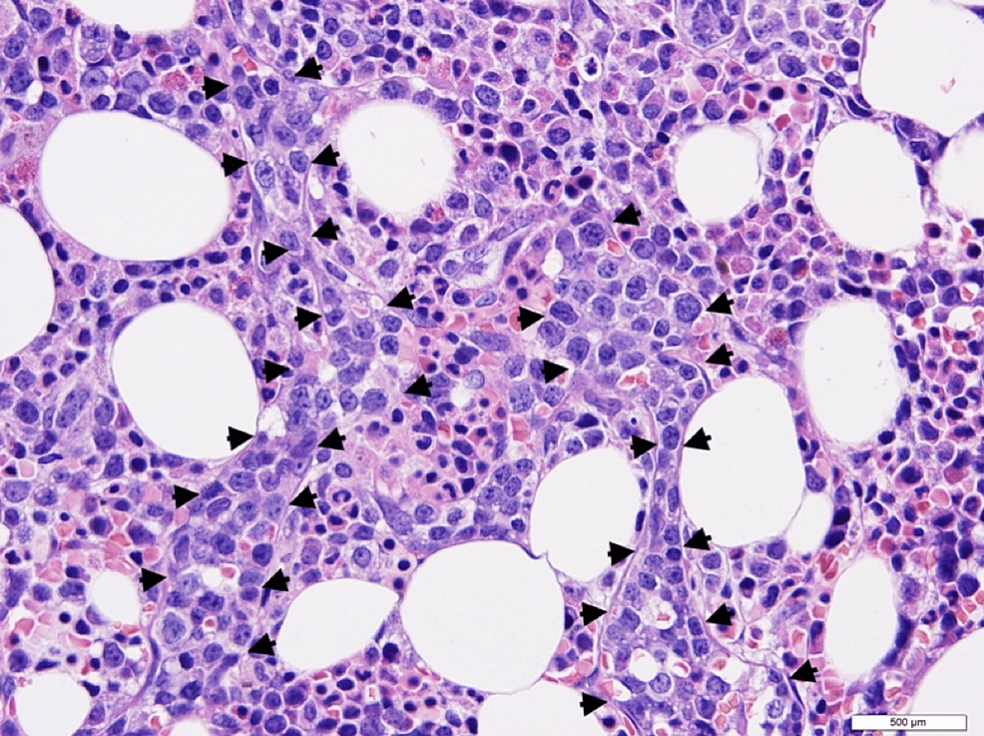
Hematoxylin and eosin section of the bone marrow under 60x magnification revealing hypercellular bone marrow with distended sinuses (arrowhead highlighting the dilated sinuses filled with large lymphoma cells)

**Figure 3:**
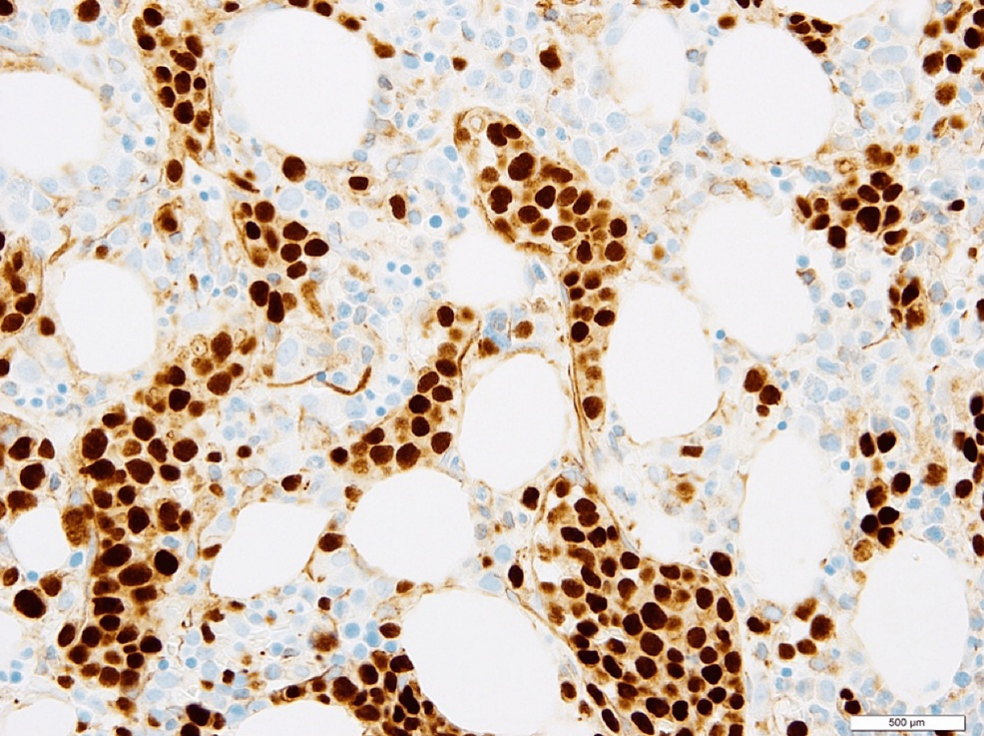
Immunohistochemical stain for PAX5 (pan-B cell marker) highlighting the intrasinusoidal lymphoma infiltrate under 60x magnification

Bone marrow pathology revealed markedly hypercellular marrow for age with extensive intrasinusoidal involvement by CD20-positive large lymphoma cells that comprised 50-60% of the marrow cellularity, confirming the diagnosis of IVLBCL (Figure 2). The lymphoma cells, by immunohistochemistry, were additionally positive for CD5, B-cell lymphoma (BCL)-2, partial BCL-6, partial cellular myelocytomatosis (c-MYC), and multiple myeloma oncogene-1 (MUM.1) with elevated Ki-67 proliferation index (Figure 3). CD31 and CD34 highlighted the marrow sinusoids filled with lymphoma cells. Fluorescence-in situ hybridization testing on marrow aspirate showed MYC and BCL-6 rearrangements and gain of one BCL-2 signal. Immunoglobulin heavy chain (IGH)-BCL2 fusion was negative. Conventional karyotype revealed an abnormal complex karyotype. A concurrent liver biopsy also showed extensive intrasinusoidal lymphoma involvement with complete obliteration of all portal tracts by the lymphoma infiltrate. Additionally, iron stains demonstrated diffuse severe hepatocyte siderosis (grade 2-3).

While awaiting pathologic confirmation, the patient had rapid deterioration of his clinical condition and a decision was made to start empiric steroids with allopurinol prophylaxis with plans to start definitive treatment with chemoimmunotherapy. However, the patient had worsening acidosis requiring intubation, and four pressors were soon started to maintain his blood pressure. The patient continued to deteriorate while attempting to initiate therapy and went into cardiac arrest. He subsequently died.

## Discussion

Due to the low incidence of IVLBCL, occurring less than one in one million individuals in the general population, and given the acute nature of generalized, systemic symptoms challenging antemortem diagnoses, very few large studies about IVLBCL exist [2]. At least two variants have been recognized: a Western variant that presents with skin and neurological involvement and an Asian variant with a hemophagocytic-like syndrome and involvement of multiple organs. A cutaneous-only variant has also been seen, which is typically less severe.

Unrelenting and persistent lactic acidosis was the striking abnormality in this patient. Lactic acidosis is classified as type A or B based on the underlying mechanism causing its overproduction or decreased clearance. Type A is the most common variant and it evolves from tissue hypoxia whereas type B can be due to a number of reasons like drugs (Metformin, beta-agonists, nucleoside reverse transcriptase inhibitors), toxins, Acquired immunodeficiency syndrome (AIDS), malignancy, thiamine deficiency, and diabetic ketoacidosis among others. In our patient, the underlying lymphoma was likely driving the lactic acidosis. Suggested mechanisms of lactic acidosis in malignancy include the Warburg effect wherein there is a preferential lactic fermentation over the oxidative phosphorylation pathway irrespective of tissue oxygenation or due to decreased lactate clearance secondary to liver involvement [3]. Warburg effect is more frequently observed in hematologic malignancies with a high proliferative index and is associated with a poor prognosis.

Interestingly, in the Asian variant of IVLBCL, there is an associated hemophagocytic component which includes hepatosplenic involvement and cytopenia. Hemophagocytic lymphohistiocytosis (HLH) secondary to IVLBCL can present with a wide variety of symptoms and lab abnormalities, but is most commonly seen with fever, bicytopenia, splenomegaly, and ferritin greater than 500 mcg/L, and can be confirmed at times with biopsy showing hemophagocytosis in the setting of the IVLBCL diagnosis [4,5].

The severity of this patient’s presentation and the rapid clinical decline in this specific patient is likely also related to secondary HLH. HLH diagnosis is based on the modified criteria published by Filipovich et al., which includes either a molecular diagnosis of hemophagocytic lymphohistiocytosis or X- linked lymphoproliferative syndrome (XLP) OR at least three of the following four: fever, splenomegaly, cytopenias (minimum two cell lines reduced), hepatitis AND at least one of four: hemophagocytosis, elevated ferritin, elevated sIL2Rα, absent or very decreased NK function. Other results that support HLH diagnosis include hypertriglyceridemia, hypofibrinogenemia and hyponatremia [6,7]..

Of the criteria mentioned, our patient had splenomegaly, elevated ferritin, bicytopenia, and fever which meets the diagnostic burden for HLH. The diagnosis of HLH, if suspected, should be followed up with natural killer cells (NK) function assay, which can show decreased NK cell function [7].

## Conclusions

We reported a case of IVLBL presenting with vague symptoms and conspicuous lactic acidosis. Swift recognition of alternative causes of unyielding lactic acidosis is essential as this condition is associated with a rapid clinical decline. Faster identification of unexplained B symptoms, cytopenias, organomegaly, and systemic inflammation can be suggestive of both IVLBCL and secondary HLH which, when identified early, will hopefully lead to early treatment initiation and decreased mortality.

## References

[1] Sillos EM, Shenep JL, Burghen GA, Pui CH, Behm FG, Sandlund JT (2001). Lactic acidosis: a metabolic complication of hematologic malignancies: case report and review of the literature. Cancer.

[2] Rajyaguru DJ, Bhaskar C, Borgert AJ, Smith A, Parsons B (2017). Intravascular large B-cell lymphoma in the United States (US): a population-based study using surveillance, epidemiology, and end results program and National Cancer Database. Leuk Lymphoma.

[3] Brault C, Zerbib Y, Delette C, Marc J, Gruson B, Marolleau JP, Maizel J (2018). The Warburg effect as a type B lactic acidosis in a patient with acute myeloid leukemia: a diagnostic challenge for clinicians. Front Oncol.

[4] Rajah FT, Al Saleh AS, Bin Salih SA (2019). Type B lactic acidosis in adults with hemophagocytic lymphohistiocytosis. Blood.

[5] Lee H, Kim HS, Lee JM (2019). Natural killer cell function tests by flowcytometry-based cytotoxicity and IFN-γ production for the diagnosis of adult hemophagocytic lymphohistiocytosis. Int J Mol Sci.

[6] Chandra H, Chandra S, Kaushik R, Bhat N, Shrivastava V (2014). Hemophagocytosis on bone marrow aspirate cytology: single center experience in north himalayan region of India. Ann Med Health Sci Res.

[7] Filipovich AH (2009). Hemophagocytic lymphohistiocytosis (HLH) and related disorders. Hematology Am Soc Hematol Educ Program.

